# Constitutive Androstane Receptor-Mediated Inhibition of Metformin on Phase II Metabolic Enzyme SULT2A1

**DOI:** 10.1155/2021/8867218

**Published:** 2021-02-15

**Authors:** Xiaowen Hu, Mengsiyu Li, Chunxue Zhang, Shuguang Pang

**Affiliations:** ^1^Department of Endocrinology, Jinan Central Hospital, Cheeloo College of Medicine, Shandong University, Jinan, China; ^2^Department of Infectious Diseases, Jinan Central Hospital, Cheeloo College of Medicine, Shandong University, Jinan, China; ^3^Department of Ultrasound, Jinan Central Hospital Affiliated to Shandong First Medical University, Jinan, China; ^4^Department of Nuclear Medicine, Jinan Central Hospital Affiliated to Shandong First Medical University, Jinan, China; ^5^Department of Endocrinology, Jinan Central Hospital Affiliated to Shandong First Medical University, Jinan, China

## Abstract

**Background:**

Metformin, as a first-line treatment for diabetes, interacts with many protein kinases and transcription factors which affect the expression of downstream target genes governing drug metabolism. Sulfotransferase, SULT2A1, one phase II metabolic enzyme, sulfonates both xenobiotic and endobiotic compounds to accelerate drug excretion. Herein, we designed experiments to investigate the effects and mechanisms of metformin on SULT2A1 expression in vitro.

**Methods:**

The hepatocellular carcinoma cell line, HepaRG, was cultured with different concentrations of metformin. The cell viability was measured using CCK8 kit. HepaRG was used to evaluate the protein expression of pregnane X receptor (PXR), the constitutive androstane receptor (CAR), SULT2A1, AMP-activated protein kinase (AMPK), and phosphorylation of AMPK (p-AMPK), respectively, at different concentrations of metformin with or without rifampin (human PXR activator) and CITCO (human CAR activator). The coregulators with CAR on SULT2A1 promoter response elements have also been characterized.

**Results:**

We showed that metformin did not affect the basic expression of SULT2A1 but could suppress the expression of SULT2A1 induced by the activator of human CAR. Investigations revealed that metformin which could block CAR nuclear translocation further suppress SULT2A1. In addition, we found that the prevented CAR transfer into the nucleus by metformin was partially an AMPK-dependent event.

**Conclusion:**

The present study indicated that the activation of AMPK-CAR pathway mediated the suppression of SULT2A1 by metformin. Metformin may affect the metabolism and clearance of drugs which are SULT2A1 substrates. The results that emerged from this work provide substantial insights into an appropriate medication in the treatment of diabetes patients.

## 1. Introduction

Metformin is a biguanide derivative that belongs to a class of oral hypoglycemic agents. It is now believed that metformin could exert a variety of biological effects. Metformin has been used worldwide not only as a classical antidiabetes medication but also for the treatment of polycystic ovarian syndrome, metabolic syndrome, nonalcoholic fatty liver disease, and other conditions [1, 2]. Besides, metformin has become the focus of intensive research as a potential anticancer agent [3, 4]. Metformin is often used in combination with other drugs. Considering the broad clinical application of metformin, drug-drug interactions (DDIs) or drug-induced adverse effects associated with metformin-based polypharmacy treatment for patients should be thoroughly evaluated [5].

In the human body, the interactions between drugs are mediated by kinds of enzymes. Concerning the influence of metformin as an initiator on the metabolism and clearance of other coadministered drugs, the effect of metformin with various enzymes is important and should be judiciously investigated. Metabolic enzymes such as cytochrome P450s (CYPs) and sulfotransferases (SULTs) are coordinately induced to eliminate drugs and their metabolites from the body to counter toxicity. Several reports have indicated that metformin can affect the pharmacokinetics of coadministered drugs metabolized by CYPs such as topiramate, dofetilide, phenprocoumon, and itraconazole [6, 7]. However, very few studies have paid attention to the interaction between metformin with SULTs so far. SULTs are one of the main phase II drug-metabolizing enzymes (DMEs), which catalyze the sulfonation of endogenous and exogenous hydroxyl-containing compounds. SULTs play a key role in a number of vital biological pathways. Sulfate conjugation is important in metabolism, increasing both xenobiotic and endobiotic water solubility to accelerate excretion [8]. To summarize, SULTs could alter the metabolism of some drugs by improving their water solubility.

SULT2A1, a member of the SULTs subfamily, shows enriched expression in the liver and adrenal gland [9]. Previous work indicated the expression of human or rodent SULT2A1 was transcriptionally regulated by nuclear receptors (NRs), mainly by the pregnane X receptor (PXR) [10] and constitutive androstane receptor (CAR) [11]. NRs are transcription factors activated by both endogenous and exogenous ligands, according to control of the target gene transcription to lead to the initiation of biological responses [12]. PXR and CAR are members of the NRs subfamily. Both are identified as xenosensors and have important roles in drug metabolism and DDIs by regulating the expression of genes encoding drug-metabolizing enzymes and transporters [13, 14]. In general, PXR and CAR are initially retained in the cytoplasm; once activated by the specific ligands, such as xenobiotics, drugs, herbal supplements, vitamins, and some endobiotics, they interact with the 9-cis-retinoic acid receptor alpha (RXR*α*), another NRs member, to form a heterodimer [15]. Subsequently, the heterodimer translocates into the nucleus and then binds to the corresponding xenobiotic response elements (XREM) in the promoter regions of target genes [16]. In a word, PXR and CAR could lead to the biotransformation and disposition of related xenobiotics according to transcriptional control.

AMP-activated protein kinase (AMPK) acts as a major cellular energy sensor and is activated by an elevated AMP/ATP ratio in line with the cellular and environmental change [17, 18]. Activation of AMPK has been reported to be involved in many metformin's activities [19, 20]. A number of pathways including AMPK are also involved in the regulation of PXR/CAR and their target genes [20, 21].

Based on the above information, nuclear receptors PXR and CAR and AMPK signaling pathways are hypothesized to mediate the regulation of SULT2A1 by metformin. We designed the experiments to investigate the effect of metformin on the expression and activity of SULT2A1. The findings could make a great contribution to guide clinical metformin-based therapy combined with multiple drug use for diabetes patients.

## 2. Materials and Methods

### 2.1. Materials

Metformin, typical PXR activator rifampin (RIF), typical CAR activator 6-(4-clorophenyl) imidazo [2,1-b] 1,3-thiazole-5-carbaldehyde O-(3,4-dichlorobenzyl) oxime (CITCO), and AMPK inhibitor 6-[4-(2-piperidin-1-ylethoxy) phenyl]-3-pyridin-4-ylpyrazolo [1,5-a] pyrimidine (compound C) were purchased from Sigma-Aldrich (St. Louis, MO, USA). Antibody against SULT2A1 was purchased from Abcam (Cambridge, UK). Antibodies against PXR, CAR, RXR*α*, the peroxisome proliferator-activated receptor gamma coactivator-1 alpha (PGC1-*α*), lamin-B1, and *β*-actin were obtained from Wuhan Sanying Biotechnology (Wuhan, China). Antibodies against AMPK and phosphorylated AMPK (Thr172) were from Cell Signaling Technology (Danvers, MA, USA). Antibody against the hepatocyte nuclear factor 4 alpha (HNF4*α*) was purchased from Santa Cruz (Santa Cruz, CA, USA). Cell Counting Kit 8 (CCK8) was purchased from Beyotime Biotech Inc. (Nantong, China).

### 2.2. Cell Culture and Treatment

HepaRG cells were obtained from Millipore (Billerica, MA, USA). Cells were seeded in the presence of Williams' Medium E supplemented with 10% fetal bovine serum, 5 *μ*g/ml human insulin, 50 *μ*M hydrocortisone hemisuccinate, 100 U/ml penicillin, and 100 mg/ml streptomycin. Cells were maintained in the medium for 2 weeks and then in the medium plus 2% dimethyl sulfoxide (DMSO) for 2 more weeks in order to obtain differentiated hepatocyte-like properties. Differentiated HepaRG cells, highly expressed nuclear receptors and SULTs enzymes, were then treated with RIF (20 *μ*M), CITCO (1 *μ*M), metformin (0.1, 0.5, 1 mM), or their combination for 24 h or 48 h before the detection of mRNA and protein expression, respectively.

### 2.3. Assessment of Cell Cytotoxicity

To verify that ≥80% cells were viable following xenobiotic exposure, cell viability was evaluated using the CCK8 assay. In brief, HepaRG cells were seeded into 96-well plates at a density of 10^4^ per well. The different concentrations (0.5, 1 mM) of metformin with or without RIF (20 *μ*M) and CITCO (1 *μ*M) were added and cultured for 0, 24, 48, and 72 h, respectively. Then, CCK8 reagent was added to each well. Following incubation for 4 h at 37°C, the absorbance was measured at 450 nm with a multidetection microplate reader (Bio-Rad Laboratories, Inc., Hercules, CA, USA).

### 2.4. Quantitative Reverse Transcription-Polymerase Chain Reaction (qRT-PCR)

Total RNA was extracted with TRIzol reagent (Invitrogen, Thermo Fisher Scientific, Inc.). And then, the RNA was reversed-transcribed into cDNA with a PrimeScript RT Master Mix reagent kit (TaKaRa, Dalian, China). Aliquots of cDNA were amplified on an Applied Biosystems 7500 real-time PCR system (Applied Biosystems, Thermo Fisher Scientific, Inc., Waltham, MA, USA) using the SYBR Premix Ex Taq kit (TaKaRa, Dalian, China). The mRNA expression of target genes was expressed as fold changes after normalization to actin. Primers were synthesized by Life Technologies (Shanghai, China). Primer sequences used for PCR were as follows: SULT2A1-forward 5'-TCATCATCATGTCGGACGATTTC-3', SULT2A1-reverse 5'-CTTGGAGTGCATCAGGCAGAG-3'; *β*-actin-forward 5'-TGGCACCCAGCACAATGAA-3', and *β*-actin-reverse 5'-CTAAGTCATAGTCCGCCTAGAAGCA-3'. The cycling conditions were as follows: initial denaturation at 95°C for 30 sec, 40 cycles at 95°C for 5 sec, 60°C for 34 sec, and 95°C for 15 sec, followed by 60°C for 1 min and 95°C for 15 sec. Data were analyzed using the 2^−ΔΔCt^ method.

### 2.5. Western Blotting

The protein expression was measured by western blotting. Nuclear protein was extracted from cells according to the manufacturer's instructions of the nuclear and cytoplasmic protein extraction kit (Beyotime Biotech Inc., Nantong, China). Following drug treatment, the medium was removed, and the cells were rinsed with ice-cold PBS. After adding ice-cold RIPA buffer with protease inhibitors, the cells were scraped from the surface of the culture dish and incubated on ice for 20 min. Subsequently, the cell lysates were centrifuged at 2000× g for 10 min at 4°C and the supernatant was collected. Total protein concentration was measured using the bicinchoninic acid (BCA) method. The proteins were loaded equally on 12% SDS-PAGE and were transferred onto PVDF membranes (0.2 mm, Millipore, Billerica, MA, USA). Then, the membranes were probed with primary antibodies against SULT2A1, PXR, CAR, RXR*α*, HNF4*α*, PGC-1*α*, AMPK, p-AMPK and *β*-actin, and lamin-B1 at 4°C overnight, followed by treatment with the second antibodies for 1 h. After removal of the secondary antibodies, the membranes were then treated with an enhanced chemiluminescence (ECL) reagent (Millipore, Billerica, MA, USA), and the signals were imaged on an automated gel imaging analysis system. Image J (NIH, Bethesda, MD, USA) was used for quantification of the relative signal intensity.

### 2.6. Statistical Analysis

We used the GraphPad Prism 6.0 (GraphPad Software Inc., San Diego, CA) to perform the statistical analysis. Student's *t*-test was used to analyze differences between the two groups. One-way analysis of variance (ANOVA) with Tukey's tests was used to analyze more than two groups. All the experimental data were presented as the mean ± SEM of at least three independent experiments. *P* < 0.05 was considered to indicate a statistically significant difference.

## 3. Results

### 3.1. Cell Viability of HepaRG Cells following Exposure to Metformin

**Figure 1:**
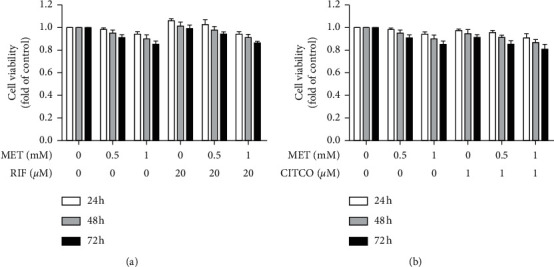
Cell viability of HepaRG cells following exposure to metformin alone and in combination with RIF or CITCO. (a) HepaRG cells were exposed to vehicle control (a similar volume of DMSO), metformin (MET; 0.5, 1 mM), RIF (20 *μ*M), or metformin in combination with RIF for 24, 48, and 72 h. (b) HepaRG cells were exposed to vehicle control (a similar volume of DMSO), metformin (0.5, 1 mM), CITCO (1 *μ*M), or metformin in combination with CITCO for 24, 48, and 72 h. Cell viability was monitored by CCK8 assay. The data shown are the mean ± SEM (*n* = 3). (^*∗*^*P* < 0.05, ^∗∗^*P* < 0.01 compared to the control group).

The cell viability test was performed to rule out any cytotoxic effects at functional doses of metformin. HepaRG cells were exposed to a range of concentrations of metformin both alone and in combination with PXR activator RIF or CAR activator CITCO for 24, 48, and 72 h. As shown in Figures 1(a) and 1(b), compared to cells treated with DMSO, metformin caused no cytotoxicity either with or without RIF/CITCO cotreatment at 48 h. Even after exposure to the highest concentration of metformin (1 mM) with RIF/CITCO at 72 h, cell viability was still approximately 80% in cells.

### 3.2. Metformin Represses CITCO-Induced SULT2A1 Expression

**Figure 2:**
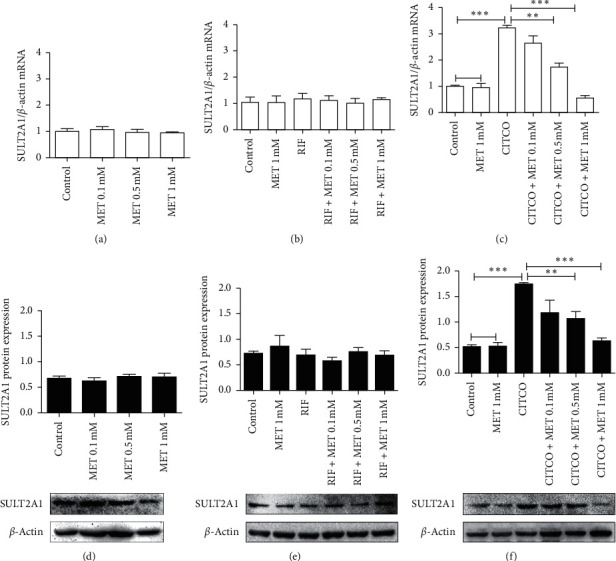
Metformin represses CITCO-induced SULT2A1 expression. (a–c) HepaRG cells were treated with RIF (20 *μ*M), CITCO (1 *μ*M), metformin (0.1, 0.5, 1 mM), or their combination for 24 h. Cells treated only with DMSO were considered as the control group. Total mRNA was collected and the expressions of SULT2A1 and *β*-actin were analyzed using real-time PCR. Values were normalized to the expression of *β*-actin. (d–f) HepaRG cells were treated with RIF (20 *μ*M), CITCO (1 *μ*M), metformin (0.1, 0.5, 1 mM), or their combination for 48 h. Whole-cell extracts were harvested, and the expression of SULT2A1 and the internal control *β*-actin were analyzed using western blotting. Quantitation of the SULT2A1 protein bands was normalized to *β*-actin. A representative blot was shown and quantified by Image J software. The data shown are the mean ± SEM (*n* = 3) (^∗∗^*P* < 0.01 and ^∗∗∗^*P* < 0.002, comparison indicated by brackets. ns: not significant).

To further evaluate the effects of metformin on SULT2A1 mRNA and protein expression, HepaRG cells were incubated with various metformin concentrations either alone or in combination with RIF/CITCO, and mRNA and proteins were extracted for further analysis. Metformin alone, no matter in what concentrations, did not affect the basal mRNA and protein of SULT2A1 levels compared with the control (Figures 2(a) and 2(d)). 20 *μ*M RIF alone also did not influence the expression of SULT2A1 mRNA and protein compared with the control (Figures 2(b) and 2(e)). 1 *μ*M CITCO significantly elevated the expression of SULT2A1 mRNA, compared with control. The lowest concentration, 0.1 mM metformin cotreatment with CITCO decreased SULT2A1 mRNA levels slightly compared with the CITCO group but was not statistically significant. Incubation of cells with 0.5 mM, 1 mM metformin cotreatment for 24 h resulted in significantly reduced SULT2A1 compared with the CITCO group (*P* < 0.01, *P* < 0.001; Figure 2(c)). Consistent with mRNA levels, overexpression of SULT2A1 protein induced by CITCO was also significantly suppressed upon metformin cotreatment (*P* < 0.01, *P* < 0.001; Figure 2(f)).

### 3.3. The Effects of Metformin with or without CITCO on CAR Protein Expression

**Figure 3:**
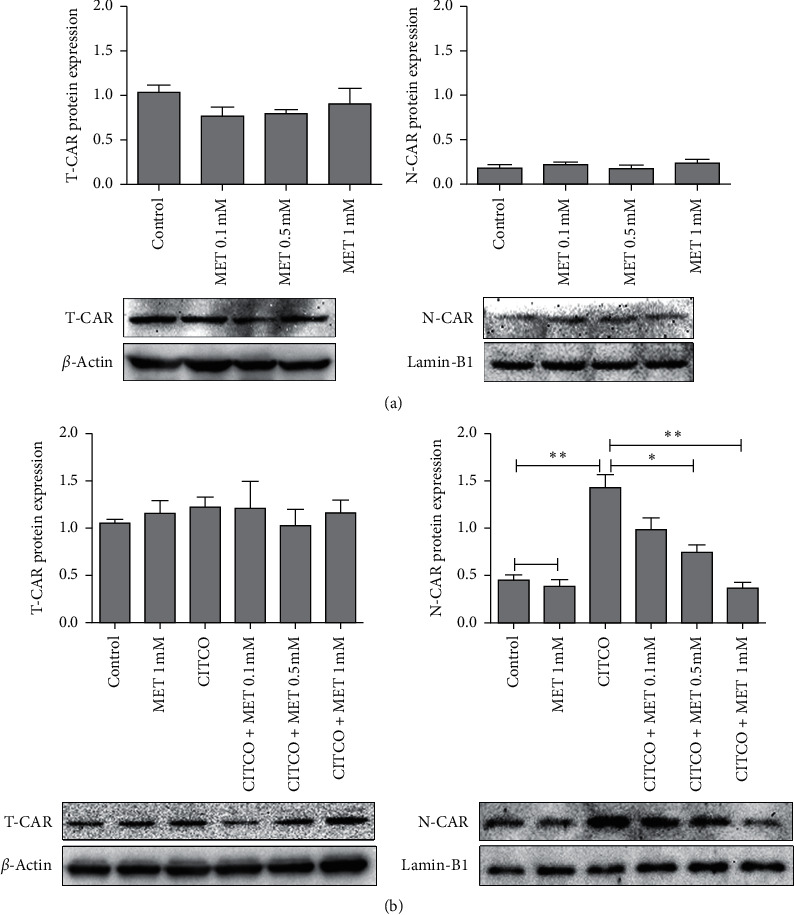
The effects of metformin with or without CITCO on CAR protein expression. HepaRG cells were treated with metformin (0.1, 0.5, 1 mM) (a) and CITCO (1 *μ*M) (b) either individually or in combination for 48 h. Cells treated only with DMSO were considered as the control group. Total cell proteins and the nuclear proteins were harvested, respectively. Protein levels were determined by western blotting and were quantified with Image J. Quantitation of the T-CAR protein bands was normalized to *β*-actin; quantitation of the N-CAR protein bands was normalized to lamin-B1. The data shown are the mean ± SEM (*n* = 3) (^*∗*^*P* < 0.05 and ^∗∗^*P* < 0.01, comparison indicated by brackets. ns: not significant).

CAR is predominantly expressed in the cytoplasm under basal condition and translocates into the nucleus elicited by activators. Nuclear translocation of CAR has been regarded as the requisite and initial step of CAR activation [22]. CITCO-treated cells increased the CAR protein in nucleus (N-CAR) but did not alter the expression of total CAR protein (T-CAR) compared with the control group. This is consistent with previous findings. CITCO is a well-known classical ligand for CAR, which can promote CAR nuclear transfer. Metformin alone did not change the expression of T-CAR and the N-CAR compared with control (Figure 3(a)). This showed that metformin alone could not promote nuclear translocation of CAR. Metformin cotreatment with CITCO also did not change the expression of T-CAR protein. However, CITCO-mediated increased N-CAR was reduced due to the addition of metformin treatment (Figure 3(b)). It implied that CITCO-mediated CAR nuclear transfer was efficiently restored by metformin.

### 3.4. The Effects of Metformin on RXR*α*, HNF4*α*, and PGC-1*α* Expression in HepaRG Cells

**Figure 4:**
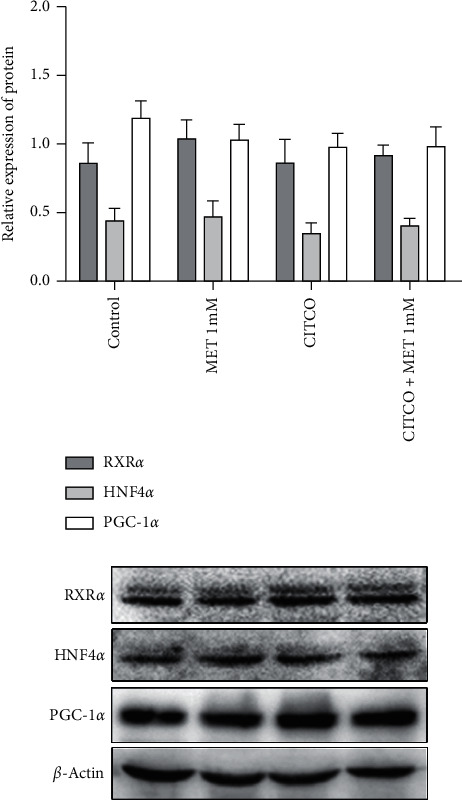
The effects of metformin on the levels of RXR*α*, HNF4*α*, and PGC-1*α* in HepaRG cells. HepaRG cells were treated with metformin (1 mM) and CITCO (1 *μ*M), either individually or in combination for 48 h. Cells treated only with DMSO were considered as the control group. Whole-cell extracts were harvested, and the expressions of RXR*α*, HNF4*α*, PGC-1*α,* and the internal control *β*-actin were analyzed using western blotting. Quantitation of the three protein bands was normalized to *β*-actin, respectively. A representative blot was shown and quantified by Image J software. The data shown are the mean ± SEM (*n* = 3).

RXR*α*, HNF4*α*, and PGC-1*α* are reported to be the three important proteins in the ligand-dependent CAR pathway [23]. Therefore, we evaluated whether the presence of metformin altered the expressions of these coregulators. Results showed no significant change in the protein expressions of RXR*α*, HNF4*α*, and PGC-1*α* following metformin with or without the presence of CITCO treatment compared with control (Figure 4).

### 3.5. Regulation of CAR and SULT2A1 by Metformin Is Dependent on the Activation of AMPK Pathways

**Figure 5:**
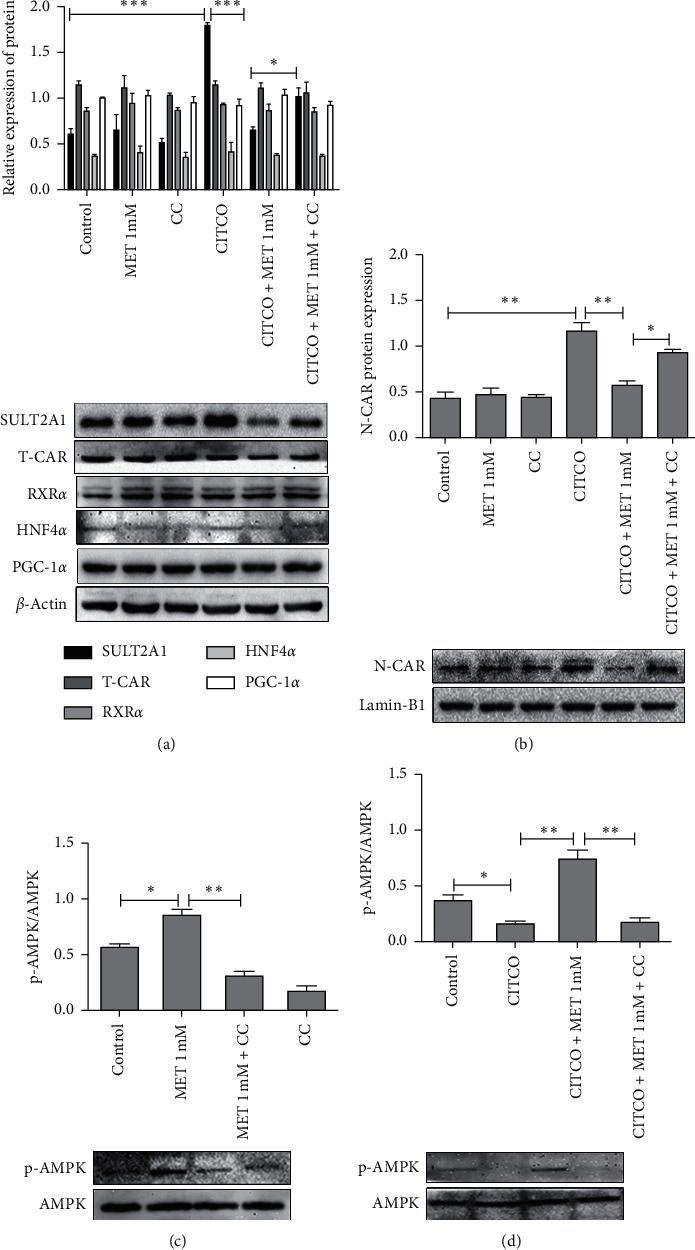
Regulation of CAR and SULT2A1 by metformin is dependent on the activation of AMPK pathways. HepaRG cells were preincubated with compound C (CC; 20 *μ*M) for 90 min before the treatment with metformin (1 mM), CITCO (1 *μ*M), or their combinations. Cells treated only with DMSO were considered as the control group. Cells were then lysed and analyzed for western blotting against SULT2A1, T-CAR, RXR*α*, PGC-1*α*, HNF4*α*, *β*-actin (a), N-CAR, lamin-B1 (b), AMPK, and p-AMPK (c, d). A representative blot was shown and quantified by Image J software. The data shown are the mean ± SEM (*n* = 3) (^*∗*^*P* < 0.05, ^∗∗^*P* < 0.01, and ^∗∗∗^*P* < 0.001, comparison indicated by brackets).

As metformin has been reported to be a well-established activator of AMPK, the roles of AMPK signaling pathways were investigated to gain insight into the underlying mechanism of metformin-CAR-SULT2A1 pathway. We used 20 *μ*M compound C, a known inhibitor of AMPK, to preincubation of the cells to block AMPK activation. Compound C alone did not affect the basal protein of SULT2A1 and N-CAR levels compared with control. CITCO-induced SULT2A1 proteins were significantly repressed by metformin. However, the metformin-mediated repression of SULT2A1 induced by CITCO was partially restored by compound C (Figure 5(a)). Similarly, metformin repressed N-CAR induced by CITCO. Compound C dramatically reversed this blocking effect (Figure 5(b)). The changing trends of SULT2A1 and N-CAR were corresponding in each group. To further verify the involvement of the pathways, phospho-lysates were prepared to detect the activation status of the respective pathway. Metformin increased the phosphorylation of AMPK (p-AMPK) and the enhanced phosphorylation of AMPK was efficiently inhibited by the presence of compound C (Figure 5(c)). CITCO decreased the phosphorylation of AMPK and the reduced phosphorylation of AMPK was efficiently increased by metformin. But this raised effect of metformin was restored by compound C (Figure 5(d)). SULT2A1 and N-CAR were inversely related to the phosphorylation of AMPK. Additionally, the expression level of T-CAR, RXR*α*, HNF4*α*, and PGC-1*α* exhibited no difference in each group (Figure 5(a)).

## 4. Discussion

Our study identified that metformin, a widely used antidiabetic drug, suppressed SULT2A1 expression by inhibiting the translocation of CAR not PXR in human hepatocytes. Our investigation also demonstrated that metformin inhibited CAR nuclear translocation mostly through the AMPK pathways. Additionally, metformin only affected changed SULT2A1 but did not affect the basal expression of SULT2A1.

The HepaRG cell line is terminally differentiated hepatocytes deriving from an Edmonson grade I differentiated hepatocellular carcinoma. Because of retaining many characteristics of primary hepatocytes, HepaRG cells are considered as the best surrogate for human hepatocytes. Unlike HepG2 and other hepatocytes, HepaRG cells exhibit properties of adult human hepatocytes, such as expressing the xenobiotic-metabolizing enzymes, transporters, and the transcription factors of controlling hepatocellular gene expression [24, 25]. Therefore, we used HepaRG cells to assess a series of experiments.

All NRs share common sequence features. They are ligand-dependent transcription factors and could regulate the transcription of their target genes by sensing both intracellular and extracellular signals. In the presence of ligand, NRs are known to shuttle continually between the cytoplasm and the nucleus to maintain an equilibrium [12]. These subsets of NRs, protecting the body from harmful toxicants, are termed xenobiotic receptors (XRs). XRs have been reported to have the ability to coordinate an array of drug metabolizing enzymes and transporters in response to endogenous and exogenous stimuli. CAR and PXR, referred to as a “master regulator” or “xenosensor,” have received the most attention. They not only regulate the expression of proteins important to drug metabolism and clearance but also formulate a defensive mechanism to protect the body against xenobiotic challenges.

The mechanisms of the SULT2A1 suppression induced by metformin are further investigated in the present study by focusing on the nuclear receptor PXR and CAR. The proximal promoter of human SULT2A1 gene contains an IR2 (inverted repeat with two spacing nucleotides) and DR4 (direct repeat with four spacing nucleotides) motifs. Previous research shows that PXR binds to both IR2 and DR4 motifs to activate this gene. CAR can also activate the SULT2A1 promoter by binding to either IR2 or DR4 [26, 27].

PXR remains repressed in terms of its gene transcription when there is no ligand. Once activated by the ligand, PXR translocates into the nucleus and binds to response sequences, thereby activating the transcription of targeted genes. RIF is one of its strong ligands [28]. Over the decades, the regulatory effect of PXR on SULT2A1 in humans is controversial [26, 29, 30]. It has been reported that PXR repressed the SULT2A1 promoter in HepG2-based assays in vitro. Echchgadda et al. showed PXR could activate the SULT2A1 expression in human colon adenocarcinoma cells [26]. However, Fang et al. showed that RIF treatment-induced SULT2A1 in only 12 out of 23 primary hepatocytes [30]. Other primary hepatocytes either repressed SULT2A1 or did not alter levels. In our studies, RIF alone did not change the expression of SULT2A1, and metformin cotreatment with RIF has no effect on SULT2A1 in HepaRG cells (Figures 2(b) and 2(e)). Our data draw a conclusion that RIF activated PXR does not involve in the metformin-SULT2A1 pathway in human hepatocytes.

We next examined whether CAR mediated the effect of metformin on SULT2A1. Similar to PXR, CAR is retained in the cytoplasm. The translocation of CAR from cytoplasm to nucleus is the initial and essential step for its activation [22]. Once CAR translocates to the nucleus, it will heterodimerize with other cofactors to regulate its target genes expression [31]. CITCO is one classic ligand for CAR. CITCO cannot affect the expression of total CAR protein levels in cells. CITCO just promotes CAR nuclear transfer resulting in a dynamic change of the distribution of CAR in the cytoplasm and nucleus. CITCO did not change T-CAR expression but increased N-CAR expression in our studies (Figure 3(b)). It further confirms the previous conclusion. Metformin alone did not change the expression of T-CAR and N-CAR expression, suggesting that metformin alone not only did not affect the total CAR protein expression but also did not change the distribution of the CAR in cells. Metformin cotreatment with CITCO did not alter the expression of T-CAR expression. However, metformin cotreatment decreased the CITCO-elevated N-CAR (Figure 3(b)). Collectively, metformin could successfully block the nuclear translocation of CAR caused by the ligand.

Correspondingly, metformin alone did not change SULT2A1 expression (Figures 2(a) and 2(d)). In contrast, metformin in combination with CITCO dramatically repressed CITCO-induced SULT2A1 expression (Figures 2(c) and 2(f)). Decreased N-CAR correlated well with the marked SULT2A1 reduction. Metformin robustly repressed ligand-induced SULT2A1 expression by reducing N-CAR. The data indicated that metformin regulated SULT2A1 according to inhibiting the ligand-induced translocation of CAR. This finding gave us an explanation that metformin suppressed CAR-mediated expression of SULT2A1, which was similar to a previous work that metformin dramatically suppressed CAR-mediated expression of CYP2B6 in human primary hepatocytes [20]. Given that CITCO and RIF are the prototypical activators of CAR and PXR, respectively, these results suggested that a most likely CAR-dependent mechanism was involved in the downregulation of SULT2A1 by metformin. However, it still deserves a thorough investigation on the metformin-CAR-SULT2A1 pathway, which should be elucidated in future studies.

Translocation of CAR into the nucleus is essential but not sufficient for the activation of its receptor. CAR and RXR*α* form a heterodimer in the cytoplasm and then transfer into the nucleus [32]. CAR-RXR*α* recruits coactivators to stimulate target gene transcription. The HNF4*α* is a central mediator of hepatocyte differentiation and lipid homeostasis in the liver. PGC-1*α* is another key transcriptional coactivator. Emerging evidence pointed that HNF4*α*, PGC-1*α,* and CAR-RXR*α* came together and enhanced the expression of various CAR target genes including SULT2A1 [23]. Results showed no significant change in the protein expressions of RXR*α*, HNF4*α,* and PGC-1*α* following metformin with or without the CITCO treatment (Figure 4). Our studies also showed that metformin decreased CITCO-mediated SULT2A1 protein expression without changing the basal expression of upper control elements (Figure 5(a)). The findings suggested that metformin suppressed CAR activation not by affecting coregulatory proteins.

AMPK activation has been known to play a central role in metformin's actions as well as in the repressed transcriptional expression of CAR-targeted genes [20, 33]. Yang et al. demonstrated that metformin robustly repressed CITCO-induced CYP2B6 expression partly through the AMPK-dependent signaling pathway. We showed in our current study that AMPK activation was also involved in the metformin-CAR-SULT2A1 pathway in HepaRG cells. CITCO notably decreased AMPK phosphorylation leading to the translocation of CAR into the nucleus. This was consistent with previous reports. The nuclear transfer of CAR was in line with the increase of N-CAR, which promoted the expression of SULT2A1. Metformin increased the phosphorylation of AMPK reduced by CITCO. The enhanced phosphorylation of AMPK inhibited the translocation of CAR and resulted in the decrease of N-CAR, which in turn repressed CITCO-induced SULT2A1 expression. Moreover, this suppression was partially recovered by the AMPK inhibitor compound C (Figure 5). Taken together, these data indicated that metformin repressed CITCO-induced SULT2A1 in human hepatocytes in part through the AMPK-dependent signaling pathway. Meanwhile, we do realize that AMPK signaling alone is not sufficient to cope with the metformin-mediated obvious suppression of SULT2A1 induction, suggesting additional signaling molecules may contribute to this specific metformin response, which warrants further investigation.

In conclusion, our results validated that metformin could repress the expression of SULT2A1 through the inhibition of CAR translocation. The suppression of SULT2A1 expression by metformin was at least in part on the activation of the AMPK pathway. The findings contribute to providing a broader view of drug-drug interactions in metformin-based drug combination that should be taken into account in diabetes drug therapy.

## Data Availability

The data used to support the findings of this study are included within the article.
